# It’s *Animals*’ Ten Year Anniversary

**DOI:** 10.3390/ani12010103

**Published:** 2022-01-02

**Authors:** Clive J. C. Phillips

**Affiliations:** 1 Curtin University Sustainability Policy Institute, Curtin University, Kent St., Bentley, WA 6102, Australia; clive.phillips@curtin.edu.au; 2 Institute of Veterinary Medicine and Animal Sciences, Estonian University of Life Sciences, Kreutzwaldi 1, 51014 Tartu, Estonia

About 30 years ago I had a discussion with my then head of department at Bangor University, the late Professor John Bryn Owen, about what an ideal journal would look like in our field, animal science, in the future. We decided that it should publish all relevant science that was statistically and ethically robust, free of the vested interests that pervaded many publications at that time. As I got more involved in animal welfare science, it was clear that such interests were prominent in this sector of the discipline. Closely associated with our ideal of opening up publication to all scientists, was that of opening up readership to all, not just a small group of subscribing society members and libraries. As the new century spawned a mass of internet content, it became clear that transitioning journals to being web-based could achieve both of these if a different funding model was used for journals — payments by authors not users. As well, using electronic communication for reviewing and editing, initial responses by the journal could be kept to about a couple of weeks (Animals mean 15.6 days), unheard of in the days of printed articles. In 2010 I was contacted by the newly formed publishing arm of the institute, MDPI, to start an open access journal in the animal science sector, the first of its kind and one of 25 journals initiated by the company that year. In the first article (Phillips, 2011, [1]), I outlined a prospectus for the new journal, including the argument that open access to animal science was a right for all community members, since their taxes had usually been used to pay for at least part of the research. I emphasized the importance of this approach for animals because of their importance to us, to the health of the planet and because of their own intrinsic value as part of our planetary heritage.

The early years were one of discovery and setting of standards. Endorsing the ARRIVE guidelines in 2014, establishing sections, enrolling editors, culminating in a structure set in 2018 with 18 sections, each headed by an experienced scientist, four deputy editors in chief, and 764 editorial board members. More recently we have reviewed our standards for use of animal and humans in research, culminating in a public statement of our policy (https://www.mdpi.com/about/announcements/3014, last accessed 31 December 2021). Recognition of the value of the journal by scientists came quickly and submissions grew exponentially. In 2019 the one thousandth paper was published, but it is a mark of the exceptional growth of this journal recently that annual publication is now more than 3500 papers and that nearly 8000 papers have now been published in total (Figure 1) [2]. Recognition by the publishing industry was slower, demonstrating some suspicion initially of open access journals, but eventually these came to be accepted. In 2013 Animals was indexed in Scopus, in 2015 Pubmed, and in 2017 Science Citation Index. The first impact factor (1.65) was received in 2018, and it has grown annually to now stand at 2.75 (five year impact 2.94). Four papers have now been cited over 100 times, including one published just five years ago, which was cited 227 times (https://doi.org/10.3390/ani6030021, last accessed 31 December 2021). With submissions rising and scientists paying regularly to publish their articles, the publishing company has been keen to support ventures to promote the science. In 2017, Travel Awards were introduced; in 2018, Young Investigator Awards; 2020, Outstanding Reviewer Award; and 2021, Best Cover Award. Reviewers are now rewarded for their work with vouchers that can be used against publication costs, as are editors. Reviewers are rated by editors for the quality of their reviews, so that the best reviewers can be used in assessment of manuscripts. In both 2020 and 2021 very successful International Electronic Conferences on Sustainability in Animals were held, with leading international keynote speakers. These gave young scientists an opportunity to present their work to a broad audience, and prizes were awarded to the best entries.

The success of Animals, which has fulfilled my vision of opening up animal science to all scientists and the community (not just through Animals, but also competing journals, which have nearly all moved to open access), has been a huge effort by many scientists, editors, publishing staff and others. This will have a lasting legacy for all the animal sciences, but particularly those, such as animal welfare and ethics, where the community has a strong interest. The future of animals on our planet lies in our hands, and we all need to have access to the debate.

What of the next ten years, what can we expect to see change? I would like to see more involvement of the community in the animal science reported in Animals. Viewing figures for Animals articles make for interesting reading (Animals|Top Cited (mdpi.com, last accessed 31 December 2021)), with those positing new ways of thinking about our relationships with animals being particularly commonly viewed. For example, the paper referred to above as being cited 227 times, which was on new ways of describing animal welfare, has been viewed over 27,000 times. This is a big impact, but we could do more. Allowing open comment on papers would enhance people’s engagement with the work and generate new ideas. As well, viewing and even commenting is one thing, actually doing something in response to new findings is much more impressive. I would like to see updates added to papers by the authors to demonstrate what outcome the article has had on public policy, animal conservation, animal welfare standards etc.

Publications in Animals are now increasingly used as the basis for online news media, including opinion piece outlets. It is vitally important that these rely on sound science, such as that reported in Animals. It was partly for this reason that I initiated the ‘Simple Summary’, for public understanding of the science. I would like to see all our papers with top quality Simple Summaries. Scientists can get help from the media staff in their institutions if necessary. One of the most common failings of authors of our articles is an apparent inability to provide a short summary suitable for public digestion.

I would like to continue to work towards more common standards in many aspects of publishing animal science, ethical standards, the quality of reviews, and scientific writing standards. Both authors and readers have a right to expect fair treatment, of the animals and humans involved in research, and of the research process generally. As well, we must weed out any false reporting, deliberate or otherwise, or weak science, so that our journal can be relied upon as the most accurate source of animal science articles.

Finally, on behalf of the animals on our planet, I must thank all those that have made Animals the success that it now is, with much hard work over the last ten years, and I look forward to seeing its development over the next ten years.

## Figures and Tables

**Figure 1:**
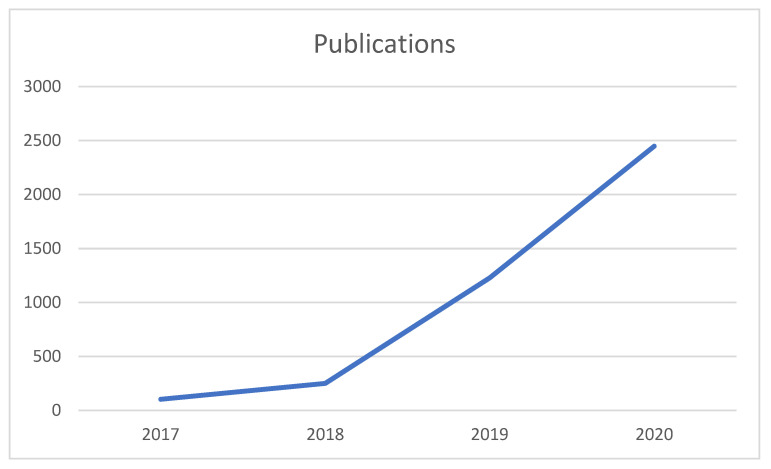
Publications from 2017 to 2020 in *Animals*.

## Data Availability

Data referred to above are available via the Animals website.
